# Pathogenic monoallelic variants in *GLIS3* increase type 2 diabetes risk and identify a subgroup of patients sensitive to sulfonylureas

**DOI:** 10.1007/s00125-023-06035-x

**Published:** 2023-12-05

**Authors:** Sarah Meulebrouck, Victoria Scherrer, Raphaël Boutry, Bénédicte Toussaint, Emmanuel Vaillant, Aurélie Dechaume, Hélène Loiselle, Beverley Balkau, Guillaume Charpentier, Sylvia Franc, Michel Marre, Morgane Baron, Martine Vaxillaire, Mehdi Derhourhi, Mathilde Boissel, Philippe Froguel, Amélie Bonnefond

**Affiliations:** 1 Inserm UMR1283, CNRS UMR8199, European Genomic Institute for Diabetes (EGID), Université de Lille, Institut Pasteur de Lille, Lille University Hospital, Lille, France; 2 Inserm U1018 Clinical Epidemiology, Center for Research in Epidemiology and Population Health, Paris-Saclay University, Paris-Sud University, UVSQ, Villejuif, France; 3 CERITD (Centre d’Étude et de Recherche pour l’Intensification du Traitement du Diabète), Evry, France; 4 Department of Diabetes, Sud-Francilien Hospital, Paris-Sud University, Corbeil-Essonnes, France; 5 Institut Necker-Enfants Malades, Inserm, Université de Paris, Paris, France; 6 Clinique Ambroise Paré, Neuilly-sur-Seine, France; 7 Department of Metabolism, Digestion and Reproduction, Imperial College London, London, UK

**Keywords:** ACMG, Functional genetics, GLIS3, Luciferase assays, Rare variants, Type 2 diabetes

## Abstract

**Aims/hypothesis:**

*GLIS3* encodes a transcription factor involved in pancreatic beta cell development and function. Rare pathogenic, bi-allelic mutations in *GLIS3* cause syndromic neonatal diabetes whereas frequent SNPs at this locus associate with common type 2 diabetes risk. Because rare, functional variants located in other susceptibility genes for type 2 diabetes have already been shown to strongly increase individual risk for common type 2 diabetes, we aimed to investigate the contribution of rare pathogenic *GLIS3* variants to type 2 diabetes.

**Methods:**

*GLIS3* was sequenced in 5471 individuals from the Rare Variants Involved in Diabetes and Obesity (RaDiO) study. Variant pathogenicity was assessed following the criteria established by the American College of Medical Genetics and Genomics (ACMG). To address the pathogenic strong criterion number 3 (PS3), we conducted functional investigations of these variants using luciferase assays, focusing on capacity of GLIS family zinc finger 3 (GLIS3) to bind to and activate the *INS* promoter. The association between rare pathogenic or likely pathogenic (P/LP) variants and type 2 diabetes risk (and other metabolic traits) was then evaluated. A meta-analysis combining association results from RaDiO, the 52K study (43,125 individuals) and the TOPMed study (44,083 individuals) was finally performed.

**Results:**

Through targeted resequencing of *GLIS3*, we identified 105 rare variants that were carried by 395 participants from RaDiO. Among them, 49 variants decreased the activation of the *INS* promoter. Following ACMG criteria, 18 rare variants were classified as P/LP, showing an enrichment in the last two exons compared with the remaining exons (*p*<5×10^−6^; OR>3.5). The burden of these P/LP variants was strongly higher in individuals with type 2 diabetes (*p*=3.0×10^−3^; OR 3.9 [95% CI 1.4, 12]), whereas adiposity, age at type 2 diabetes diagnosis and cholesterol levels were similar between variant carriers and non-carriers with type 2 diabetes. Interestingly, all carriers with type 2 diabetes were sensitive to oral sulfonylureas. A total of 7 P/LP variants were identified in both 52K and TOPMed studies. The meta-analysis of association studies obtained from RaDiO, 52K and TOPMed showed an enrichment of P/LP *GLIS3* variants in individuals with type 2 diabetes (*p*=5.6×10^−5^; OR 2.1 [95% CI 1.4, 2.9]).

**Conclusions/interpretation:**

Rare P/LP *GLIS3* variants do contribute to type 2 diabetes risk. The variants located in the distal part of the protein could have a direct effect on its functional activity by impacting its transactivation domain, by homology with the mouse GLIS3 protein. Furthermore, rare P/LP *GLIS3* variants seem to have a direct clinical effect on beta cell function, which could be improved by increasing insulin secretion via the use of sulfonylureas.

**Graphical Abstract:**

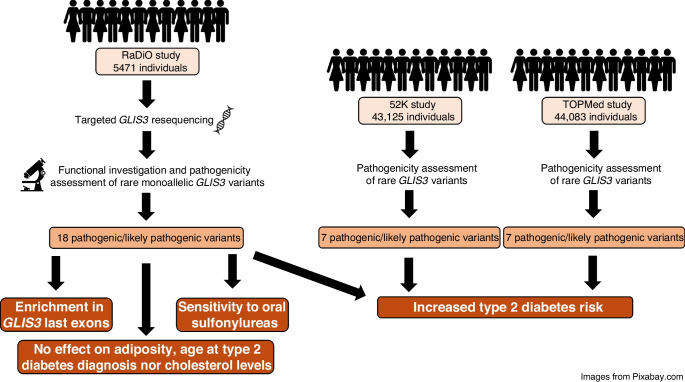

**Supplementary Information:**

The online version contains peer-reviewed but unedited supplementary material available at 10.1007/s00125-023-06035-x.


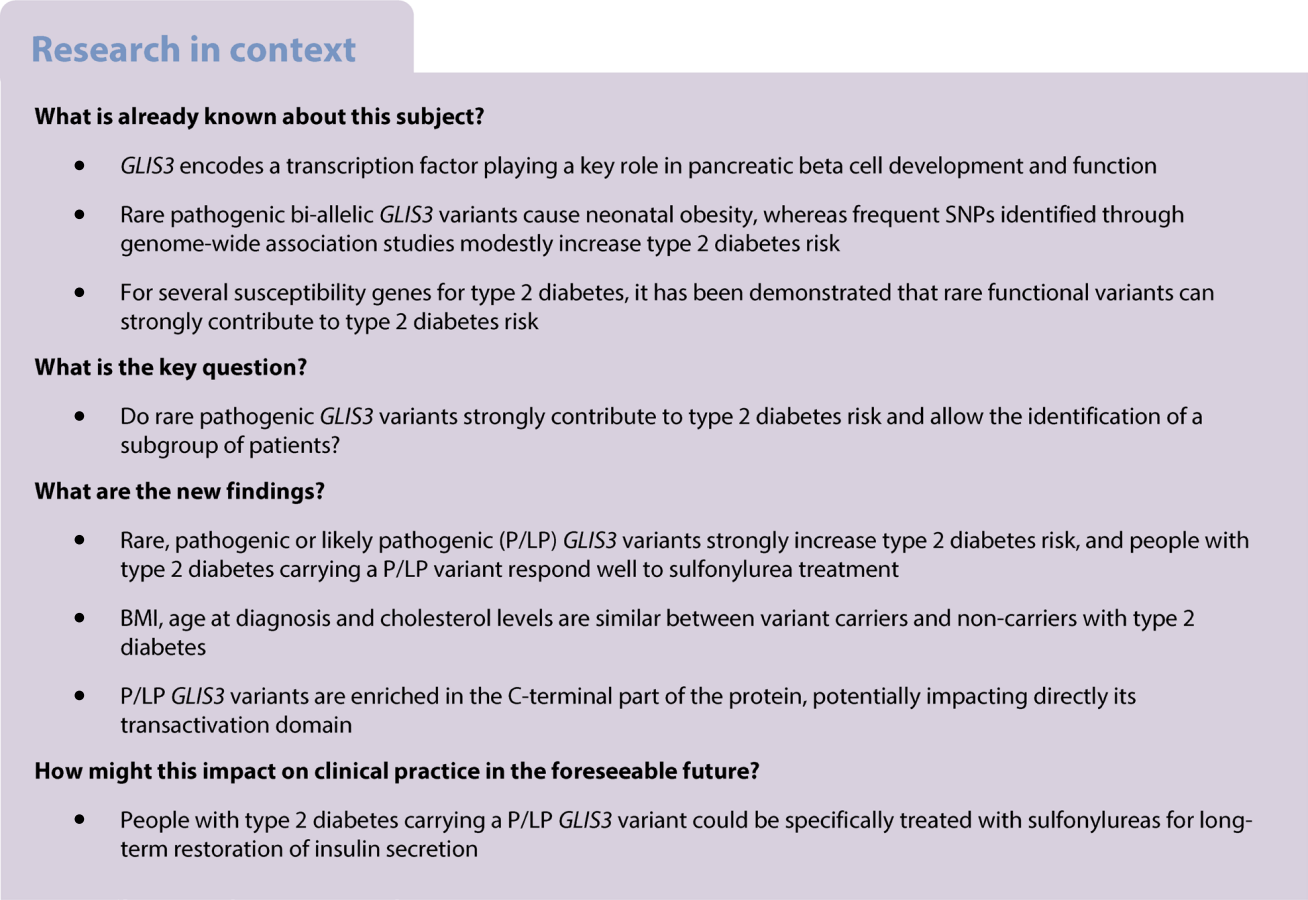


## Introduction

Type 2 diabetes, like many other multifactorial disorders, includes monogenic forms that are rare, more severe, and appear earlier in life than common polygenic forms [1]. Rare, pathogenic, bi-allelic variants in *GLIS3* cause a severe syndrome that includes neonatal diabetes [2]. GLIS family zinc finger 3 (GLIS3) is a transcription factor playing a major role in pancreatic beta cell development and function. Notably, it enhances the transcription of *INS* by binding to its promoter and recruiting the transcription factors pancreatic and duodenal homeobox 1 (PDX1), MafA and neuronal differentiation 1 (NEUROD1) [3]. In addition, genome-wide association studies (GWAS) identified frequent SNPs at the *GLIS3* locus associated with common type 2 diabetes risk, and with variation in fasting glucose and beta cell function [4]. We and others previously found strong impact on common type 2 diabetes risk of rare, functional variants located in susceptibility genes identified by GWAS [1], paving the way for new insights into the underlying pathophysiology and personalised treatment strategies. This approach is particularly fruitful as it is challenging to uncover such information solely through GWAS, where associated SNPs are typically non-coding and exhibit modest effect [5].

We wondered whether rare, deleterious *GLIS3* variants could be associated with increased risk for common type 2 diabetes, and whether they might impact drug treatment choices. In this respect, a recent study showed an association between rare *GLIS3* variants and increased disease risk [6], but the lack of pathogenicity assessment using in vitro data may limit its conclusion.

## Methods

### Study participants

We analysed 5471 blood DNA samples accurately sequenced from several population studies included in the Rare Variants Involved in Diabetes and Obesity (RaDiO) study [7]. The RaDiO study and criteria of inclusion are described in detail in the electronic supplementary material (ESM) Methods.

### GLIS3 sequencing and variant annotation

*GLIS3* DNA sequencing (NM_001042413.2) was previously performed by next-generation sequencing [7]. Only rare variants with a minor allele frequency (MAF) below 1% in any population study in the GnomAD browser (v2.1.1) and in the present study were kept for further analyses. All rare coding variants detected in *GLIS3* had a quality QUAL score higher than 50. No variant presented more than 5% of missing genotype across the participants.

### Statistical analyses for association studies

The burdens of rare coding variants identified in RaDiO were analysed as a single cluster using the mixed-effects score test (MiST), as previously described [7]. The association studies between the burdens of variants and clinical traits were adjusted for age, sex, BMI and ancestry (for assessing type 2 diabetes risk, age at type 2 diabetes diagnosis and cholesterol levels), or for age, sex and ancestry (for assessing BMI). Participant ancestry was assessed using the first five genotypic principal components as previously described [7]. The meta-analysis was performed using the generic inverse variance method from the R package meta [8]. The common effect model was applied because of low heterogeneity.

### Plasmid generation

Plasmids encoding wild-type *GLIS3* gene and rare variants were either purchased from Twist Bioscience (San Francisco, CA, USA) or generated from the first one using the Quick Change site-directed mutagenesis kit from Stratagene (San Diego, CA, USA), and verified by Sanger sequencing. Please see ESM Methods for more details.

### Culture of HEK293 cells

Human embryonic kidney 293 **(**HEK293) cells were purchased from American Type Culture Collection (Manassas, VA, USA). These cells were cultured in DMEM containing 10% FBS (vol./vol.) and 50 units/ml penicillin/streptomycin. These cells were regularly tested for mycoplasma contamination.

### Luciferase assays

HEK293 cells were transfected in suspension using Lipofectamine 2000 Transfection Reagent (Thermo Fisher Scientific, Waltham, MA, USA), with wild-type or mutated *GLIS3* plasmid, plasmid including the gene encoding firefly luciferase driven by the 5′ flanking region of *INS* containing GLIS binding sites, and plasmid including the gene encoding β-galactosidase, with or without *MAFA* plasmid. Please see ESM Methods for further details.

## Results

Through targeted resequencing of *GLIS3* (NM_001042413.2) in 5471 participants from RaDiO [7], we detected 105 rare coding *GLIS3* variants, including one nonsense variant (p.Y627*), among 395 carriers (ESM Table 1). At this stage of analysis, the burden of rare variants was not associated with type 2 diabetes risk (*p*_π_=0.20 [*p*_overall_=0.055], where *p*_π_ is the* p* value for the impact of the burden; with an OR of 0.85 [95% CI, 0.66, 1.1]; Table 1).

**Table 1:** Association analyses between rare coding *GLIS3* variants and type 2 diabetes risk

Variants	*N*	% of T2D in carriers	% of T2D in non-carriers	OR [95% CI]	*p*_π_	*p*_τ_	*p*_overall_
Rare	5356	49	39	0.85 [0.66, 1.1]	0.20	0.049	0.055
P/LP	5471	67	40	3.9 [1.4, 12]	8.0×10^−3^	0.041	3.0×10^−3^

Association analyses were performed using the MiST method for the burden of the 105 rare *GLIS3* variants, or for the burden of the 18 P/LP variants only

MiST provides a score statistic S(π) for the mean effect (π) of the cluster, and a score statistic S(τ) for the heterogeneous effect (τ) of the cluster. The overall *p* value combined *p* values *p*_π_ and *p*_τ_

*p*_π_, *p* value indicating the impact of the burden; *p*_*τ*_, *p* value indicating the heterogeneity of the burden; T2D, type 2 diabetes

To assess the pathogenicity of the 105 variants, we used the criteria from the American College of Medical Genetics and Genomics (ACMG) [9]; notably we developed in vitro assays to address the pathogenic strong criterion number 3 (PS3). Plasmids including each *GLIS3* variant were overexpressed along with their gene reporter assay to assess the ability of each mutant to bind to the 5′ flanking region of *INS* containing GLIS3 binding sites. Furthermore, these variants were evaluated in conjunction with the overexpression of MafA to determine their capacity for recruiting this transcription factor to the *INS* 5′ flanking region, consequently enhancing luciferase signalling. When compared with wild-type *GLIS3*, 49 variants decreased luciferase activity with and/or without the addition of *MAFA* in the system, and were considered loss-of-function (ESM Fig. 1).

In comparison with our functional results, the in silico pathogenicity prediction by REVEL had a poor sensitivity as only 4% of loss-of-function variants were predicted to be deleterious (ESM Table 1), but had a high specificity (ESM Table 1).

**Fig. 1:**
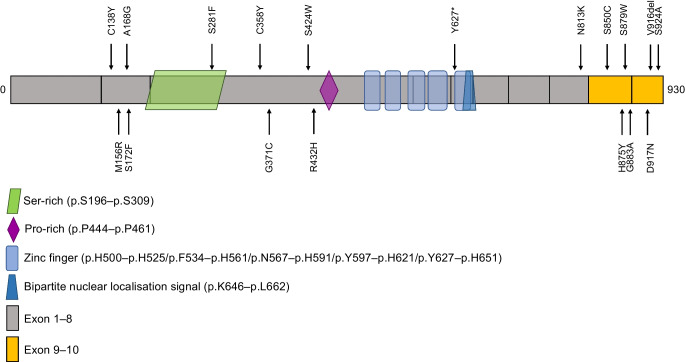
Location of the 18 P/LP variants in *GLIS3*, shown using arrows. Ser-rich, regions enriched in serine; Pro-rich, region enriched in proline

Following ACMG criteria including the PS3 criterion, 18 out of 105 variants, carried by 18 unrelated individuals of European ancestry, were found to be pathogenic or likely pathogenic (P/LP; Fig. 1 and ESM Table 2). Furthermore, P/LP variants were strongly enriched in the C-terminal part of *GLIS3*, i.e. the last two coding exons after accounting for exon length (*p*<5×10^−6^ with an OR>3.5; Fig. 1 and ESM Table 3).

We then assessed the effect of the burden of the 18 rare P/LP *GLIS3* variants on type 2 diabetes risk. This burden was four times higher among participants with type 2 diabetes compared with controls (*p*_π_=8.0×10^−3^ [*p*_overall_=3.0×10^−3^]; with an OR of 3.9 [95% CI 1.4, 12]; Table 1). We found an equal number of female and male participants carrying a P/LP *GLIS3* variant, either with type 2 diabetes or not (data not shown). Interestingly, all participants with type 2 diabetes carrying a P/LP *GLIS3* variant were treated with sulfonylureas, a class of medication that stimulates the secretion of insulin by activating ATP-dependent potassium channels. In contrast, 50% of non-carriers suffering from type 2 diabetes were treated with sulfonylureas. The sibling of one of the participants with type 2 diabetes (carrying the P/LP variant encoding p.A168G) also presented with type 2 diabetes and was also treated with sulfonylureas. The age at diagnosis, BMI and cholesterol levels were similar among P/LP variant carriers vs non-carriers with type 2 diabetes (ESM Table 4).

We then analysed the association between P/LP variants in *GLIS3* (NM_001042413 [ENST00000381971] transcript) and type 2 diabetes risk in the Type 2 Diabetes Knowledge Portal (using the genetic association interactive tool) [10]. In 43,125 individuals from 52K and in 44,083 individuals from TOPMed, we only found eight P/LP variants per study (i.e. loss-of-function transcript effect estimator [LofTee] variants with a very low MAF in GnomAD; ESM Table 5) that had no effect on disease risk due to a low statistical power (*p*=0.13 with an OR of 1.7 and *p*=0.44 with an OR of 3.3, respectively; variable threshold test). No P/LP missense variants identified in our study were observed in either TOPMed or the 52K study. However, through a meta-analysis of RaDiO, TOPMed and 52K studies, we found an enrichment of P/LP *GLIS3* variants among individuals with type 2 diabetes (*p*=5.6×10^−5^ with an OR of 2.1 [1.4, 2.9]).

## Discussion

On the basis of functional genetics, we identified 18 P/LP heterozygous *GLIS3* variants that increase type 2 diabetes risk. This corroborates the continuum between monogenic and polygenic type 2 diabetes [5], and supports the importance of functional genomics to identify variants associated with metabolic disorders, as previously demonstrated [1].

Importantly, all the participants carrying a P/LP *GLIS3* variant were treated with sulfonylureas, and their BMI was similar to non-carriers. This suggests that these variants directly alter beta cell function, but that this defect can be compensated for effectively by increasing beta cell insulin secretion. In the context of precision medicine, individuals harbouring a rare P/LP *GLIS3* variant might be deemed suitable candidates for treatment with sulfonylureas, over other medication options.

Furthermore, we observed an enrichment of P/LP variants in the C-terminal domain of GLIS3. In a prior study, two individuals with type 2 diabetes carried two highly rare deleterious variants in the last exons [6]. We suggest that mutations located at the C-terminal domain of GLIS3 could directly affect its activity by impacting its transactivation domain. Indeed, the transactivation domain of GLIS3 mouse protein, which presents a high homology with human GLIS3, is located in its C-terminal section [11], and the human c.2338dupC mutation, which generates a truncated protein (missing the last coding exons), strongly decreased GLIS3 transcriptional activity [2].

Our study has limitations, as our luciferase experiments focused on the ability of GLIS3 to bind to the *INS* promoter only, despite its multiple transactivation role in beta cells. It is thus possible that some variants impacting other aspects of GLIS3 function were not identified as loss-of-function in this study.

In conclusion, P/LP monoallelic *GLIS3* variants contribute to increased type 2 diabetes risk, in addition to *GLIS3* involvement in monogenic diabetes. Sulfonylureas might be sufficient to manage type 2 diabetes in the carriers.

### Supplementary Information

Below is the link to the electronic supplementary material.

Supplementary file1 (PDF 820 KB)

## Data Availability

Data regarding variants from TOPMed and 52K studies are available on the AMP Type 2 Diabetes Knowledge Portal via this link: https://t2d.hugeamp.org/gait.html?gene=GLIS3&tests=burden%2Cvt&transcript=ENST00000381971. Data regarding *GLIS3* variants from the GnomAD browser (v2.1.1) via this link: https://gnomad.broadinstitute.org/gene/ENSG00000107249?dataset=gnomad_r2_1 Other data are available upon request to the corresponding author.

## Abbreviations

ACMG: American College of Medical Genetics and Genomics
GLIS3: GLIS family zinc finger 3
GWAS: Genome-wide association study
HEK293: Human embryonic kidney 293
MAF: Minor allele frequency
MiST: Mixed-effects score test
P/LP: Pathogenic or likely pathogenic
PS3: Pathogenic strong criterion number 3
RaDiO: Rare Variants Involved in Diabetes and Obesity

## Author’s relationship and activities

AB is a member of the Editorial Board of *Diabetologia*. The authors declare that they have no other relationships or activities that might bias, or be perceived to bias, their work.
