# Supplementary material for: Pathogenic monoallelic variants in GLIS3 increase type 2 diabetes risk and identify a subgroup of patients sensitive to sulfonylureas
Source: Diabetologia. 2023 Dec 5;67(2):327–32. doi: 10.1007/s00125-023-06035-x (PMC10789827; doi:10.1007/s00125-023-06035-x)
Supplement: Supplementary file 1 — Supplementary file1 (PDF 820 KB) [file 125_2023_6035_MOESM1_ESM.pdf]

Electronic Supplementary Material (ESM)

Methods

Study participants. In the Rare Variants Involved in Diabetes and Obesity study (RaDiO) [1], we accurately sequenced up to 5,471 blood DNA samples from several population studies. These comprise 1/ the 9-year prospective D.E.S.I.R. study that includes middle-aged men and women from western France [2]; 2/ participants who were recruited and followed-up by the UMR 1283/8199 unit (Lille, France), by the Department of Nutrition of Hotel-Dieu Hospital (Paris, France), or by the Centre d'Etude du Polymorphisme Humain (CEPH, Saint-Louis Hospital, Paris, France) [3, 4]; 3/ participants who were recruited from Endocrinology Department of the Corbeil-Essonnes Hospital (Corbeil-Essonnes, France) [3]; 4/ the French Fleurbaix-Laventie Ville Santé study [5]. All the studies followed ethical principles defined in the Helsinki declaration (revised in 1996), and were approved by local ethical committees from Corbeil-Essonnes hospital (France), Comité Consultatif de Protection des Personnes se prêtant à des Recherches Biomédicales (CCPPRB) of Lille - Lille Hospital (Lille, France), Hotel-Dieu hospital (France), Bicêtre hospital (France). All participants to these studies signed an informed consent form. Participants with type 2 diabetes included in RaDiO had a fasting glucose ≥ 7.0 mmol l^{-1} and/or were treated for hyperglycaemia, and were negative for islet autoantibodies. Control individuals included in the study had fasting glucose <5.6 mmol l^{-1} after age 40, and were not treated for hyperglycaemia. The study population predominantly consisted of individuals of European descent, as indicated by our prior genetic analyses [1]. It is representative of the French population with type 2 diabetes, as well as the general adult population (D.E.S.I.R.). Sex was self-reported and was used as covariate in all statistical analyses. The study was not primarily designed according to sex status. Gender was not analyzed. This study did not include socioeconomic factors.

Plasmid generation. Plasmids including wild-type coding *GLIS3* gene and the five rare *GLIS3* variants encoding p.P376S, p.P684L, p.G802S, p.S879L and p.E929K were purchased from Twist Bioscience (San Francisco, California, USA). Plasmids containing the remaining variants were generated from the plasmid containing wild-type coding *GLIS3* using the Quick Change site-directed mutagenesis kit from Stratagene (San Diego, California, USA). The sequence of each plasmid was verified by Sanger sequencing.

Luciferase assays. HEK293 cells were transfected in suspension using Lipofectamine 2000 Transfection Reagent (Thermo Fisher Scientific), with 200 ng ml^{-1} of wild-type or mutated *GLIS3* plasmid, 200 ng ml^{-1} of a plasmid including the gene encoding firefly luciferase driven by the 5’ flanking region of *INS* (spanning from -423 to -18 from the transcription start site) containing the GLIS binding sites, and 50 ng ml^{-1} of a plasmid including the gene encoding beta-galactosidase, with or without 50 ng ml^{-1} of *MAFA* plasmid. 100 µl of these cells (1×10^6 cells ml^{-1}) were then seeded in a poly-L-lysine coated 96-well plate. 48 hours later, these cells were lysed in 100 µl of Luciferase Cell Culture Lysis 5X Reagent from Promega (Madison, Wisconsin, USA) for 30 minutes. Luciferase and beta-galactosidase activities were measured as previously described [6], and luciferase measurements (in relative luminescence units) were normalized by beta-galactosidase values. The experiments were performed in technical triplicate, and each experiment was reproduced at least four times. For each condition, fold-change was computed by dividing normalized luciferase activity by the mean of the wild-type *GLIS3* baseline luciferase activity (*i.e.* without MAFA). The effect of each variant was then analyzed using an ANOVA model followed by a Games-Howell post-hoc test. The mutations were analyzed in a blinded manner, meaning that the engineer conducting the analysis was unaware of the phenotype of the mutation carriers. None exclusion criteria were applied.

References

1. Bonnefond A, Boissel M, Bolze A, et al (2020) Pathogenic variants in actionable MODY genes are associated with type 2 diabetes. Nat Metab 2(10):1126–1134. https://doi.org/10.1038/s42255-020-00294-3

2. Balkau B (1996) [An epidemiologic survey from a network of French Health Examination Centres, (D.E.S.I.R.): epidemiologic data on the insulin resistance syndrome]. Rev Dépidémiologie Santé Publique 44(4):373–375

3. Sladek R, Rocheleau G, Rung J, et al (2007) A genome-wide association study identifies novel risk loci for type 2 diabetes. Nature 445(7130):881–885. https://doi.org/10.1038/nature05616

4. Meyre D, Delplanque J, Chèvre J-C, et al (2009) Genome-wide association study for early-onset and morbid adult obesity identifies three new risk loci in European populations. Nat Genet 41(2):157–159. https://doi.org/10.1038/ng.301

5. Romon M, Lafay L, Bresson JL, et al (2004) Relationships between physical activity and plasma leptin levels in healthy children: the Fleurbaix-Laventie Ville Santé II Study. Int J Obes Relat Metab Disord J Int Assoc Study Obes 28(10):1227–1232. https://doi.org/10.1038/sj.ijo.0802725

6. Baron M, Maillet J, Huyvaert M, et al (2019) Loss-of-function mutations in MRAP2 are pathogenic in hyperphagic obesity with hyperglycemia and hypertension. Nat Med 25(11):1733–1738. https://doi.org/10.1038/s41591-019-0622-0

7. Ioannidis NM, Rothstein JH, Pejaver V, et al REVEL: an Ensemble Method for Predicting the Pathogenicity of Rare Missense Variants. Am J Hum Genet. https://doi.org/10.1016/j.ajhg.2016.08.016

ESM Table 1. Rare *GLIS3* variants (NM_001042413.2) detected in the RaDiO study.

Chr	Pos (Hg19)	Mutation	MAC in gnomAD	MAF in gnomAD	MAC in RaDiO	*In silico* score[a]	LOF or neutral[b]
9	4286392	c.34C>T, p.R12W	47	0.00017	1	0.080	Neutral
9	4286362	c.64A>G, p.M22V	1	0.0000040	1	0.055	Neutral
9	4286344	c.82A>G, p.I28V	73	0.00026	2	0.018	Neutral
9	4286340	c.86C>T, p.P29L	0	0	1	0.050	Neutral
9	4286320	c.106G>A, p.G36R	33	0.00012	1	0.079	LOF
9	4286313	c.113C>G, p.P38R	1	0.0000040	1	0.22	Neutral
9	4286224	c.202A>G, p.M68V	1	0.0000040	1	0.027	Neutral
9	4286194	c.232C>G, p.R78G	154	0.00055	3	0.026	Neutral
9	4286193	c.233G>A, p.R78H	8	0.000028	1	0.013	Neutral
9	4286174	c.252A>T, p.L84F	54	0.00019	10	0.19	Neutral
9	4286170	c.256C>T, p.P86S	0	0	1	0.070	Neutral
9	4286151	c.275C>T, p.T92I	14	0.000050	1	0.093	Neutral
9	4286139	c.287C>T, p.P96L	56	0.00020	3	0.15	Neutral
9	4286068	c.358G>C, p.G120R	6	0.000021	3	0.17	LOF
9	4286056	c.370C>T, p.P124S	2	0.0000080	2	0.040	Neutral
9	4125917	c.413G>A, p.C138Y	0	0	1	0.18	LOF
9	4125908	c.422T>C, p.I141T	64	0.00023	7	0.071	LOF
9	4125905	c.425G>A, p.G142E	0	0	1	0.32	Neutral
9	4125863	c.467T>G, p.M156R	0	0	1	0.57	LOF
9	4125858	c.472G>T, p.V158F	19	0.000067	1	0.27	LOF
9	4125834	c.496C>G, p.P166A	127	0.00045	2	0.37	LOF
9	4125827	c.503C>G, p.A168G	0	0	1	0.24	LOF
9	4125815	c.515C>T, p.S172F	0	0	1	0.36	LOF
9	4118765	c.713C>G, p.S238C	1	0.0000040	1	0.17	LOF
9	4118720	c.758G>C, p.S253T	0	0	1	0.052	Neutral
9	4118685	c.793G>T, p.V265F	76	0.00027	1	0.070	Neutral
9	4118664	c.814T>A, p.Y272N	1	0.0000040	2	0.40	Neutral
9	4118651	c.827C>T, p.T276M	3	0.000012	1	0.090	LOF
9	4118645	c.833G>A, p.S278N	0	0	1	0.031	Neutral
9	4118636	c.842C>T, p.S281F	0	0	1	0.14	LOF
9	4118634	c.844C>G, p.P282A	451	0.0016	15	0.10	Neutral

9	4118588	c.890G>A, p.R297H	1	0.0000040	1	0.097	Neutral
9	4118585	c.893C>A, p.S298Y	598	0.0021	52	0.18	LOF
9	4118541	c.937G>A, p.G313R	11	0.000044	2	0.45	LOF
9	4118510	c.968C>T, p.S323L	2	0.0000080	1	0.61	LOF
9	4118504	c.974C>T, p.T325M	8	0.000028	4	0.23	LOF
9	4118466	c.1012C>T, p.P338S	0	0	2	0.21	Neutral
9	4118415	c.1063C>T, p.R355C	1	0.0000042	1	0.24	LOF
9	4118405	c.1073G>A, p.C358Y	0	0	1	0.18	LOF
9	4118393	c.1085C>A, p.P362Q	301	0.0012	4	0.063	LOF
9	4118388	c.1090C>T, p.P364S	378	0.0015	31	0.068	Neutral
9	4118367	c.1111G>T, p.G371C	0	0	2	0.082	LOF
9	4118361	c.1117C>G, p.L373V	269	0.0012	3	0.043	LOF
9	4118352	c.1126C>T, p.P376S	551	0.0025	10	0.023	Neutral
9	4118333	c.1145C>A, p.P382Q	3	0.000014	1	0.049	Neutral
9	4118324	c.1154G>T, p.G385V	41	0.00020	5	0.052	Neutral
9	4118293	c.1185G>A, p.M395I	0	0	1	0.097	Neutral
9	4118287	c.1191G>C, p.Q397H	462	0.0021	24	0.058	Neutral
9	4118279	c.1199A>G, p.H400R	175	0.00080	9	0.078	Neutral
9	4118278	c.1200C>G, p.H400Q	123	0.00056	1	0.017	Neutral
9	4118268	c.1210C>A, p.Q404K	6	0.000032	1	0.12	LOF
9	4118264	c.1214C>T, p.P405L	13	0.000058	2	0.036	LOF
9	4118262	c.1216G>T, p.G406C	628	0.0028	13	0.13	LOF
9	4118251	c.1227C>A, p.N409K	17	0.000075	1	0.063	LOF
9	4118246	c.1232T>A, p.M411K	0	0	1	0.29	Neutral
9	4118207	c.1271C>G, p.S424W	0	0	1	0.045	LOF
9	4118183	c.1295G>A, p.R432H	0	0	1	0.077	LOF
9	4118160	c.1318A>G, p.T440A	217	0.00099	3	0.011	Neutral
9	4118153	c.1325A>C, p.D442A	0	0	1	0.097	Neutral
9	4118142	c.1336G>A, p.A446T	0	0	1	0.025	Neutral
9	4118090	c.1388A>C, p.H463P	3	0.000016	2	0.19	Neutral
9	4118061	c.1417C>T, p.L473F	22	0.000088	3	0.19	LOF
9	4118048	c.1430C>T, p.A477V	192	0.00074	2	0.047	Neutral
9	4117998	c.1480G>A, p.D494N	7	0.000028	1	0.20	Neutral

9	4117995	c.1483G>A, p.G495S	1	0.0000040	1	0.083	Neutral
9	4117960	c.1518C>G, p.D506E	8	0.000028	2	0.47	LOF
9	4117933	c.1545G>C, p.E515D	820	0.0029	59	0.24	Neutral
9	4117769	c.1709C>T, p.T570M	5	0.000018	2	0.15	Neutral
9	3937185	c.1715A>T, p.E572V	1	0.0000040	1	0.71	LOF
9	3937125	c.1775C>G, p.T592R	1	0.0000040	1	0.54	LOF
9	3937057	c.1843G>A, p.A615T	1	0.0000040	1	0.35	Neutral
9	3932462	c.1881T>A, p.Y627*	0	0	1	NA	LOF
9	3898825	c.1994G>A, p.S665N	24	0.000085	1	0.052	Neutral
9	3898768	c.2051C>T, p.P684L	11	0.000039	1	0.035	Neutral
9	3898759	c.2060C>T, p.S687F	74	0.00026	5	0.23	LOF
9	3898752	c.2067A>T, p.R689S	0	0	1	0.14	Neutral
9	3898730	c.2089G>A, p.V697M	226	0.00080	4	0.039	LOF
9	3898723	c.2096G>A, p.R699H	69	0.00024	2	0.024	Neutral
9	3898712	c.2107C>T, p.P703S	7	0.000028	3	0.040	Neutral
9	3879583	c.2141C>G, p.S714C	5	0.000018	2	0.10	Neutral
9	3879443	c.2281G>A, p.D761N	2	0.000008	2	0.07	LOF
9	3856177	c.2305C>A, p.P769T	24	0.000085	2	0.019	Neutral
9	3856170	c.2312C>A, p.A771D	0	0	1	0.10	Neutral
9	3856078	c.2404G>A, p.G802S	1	0.0000040	1	0.18	Neutral
9	3856051	c.2431G>C, p.E811Q	1	0.0000040	1	0.033	LOF
9	3856043	c.2439C>G, p.N813K	0	0	1	0.013	LOF
9	3856021	c.2461A>G, p.I821V	0	0	1	0.11	Neutral
9	3829472	c.2494A>G, p.K832E	39	0.00016	1	0.071	Neutral
9	3829448	c.2518G>A, p.D840N	22	0.000078	1	0.20	LOF
9	3829435	c.2531T>C, p.I844T	12	0.000043	2	0.024	Neutral
9	3829417	c.2549C>G, p.S850C	0	0	1	0.26	LOF
9	3829357	c.2609G>A, p.S870N	1	0.000032	3	0.074	Neutral
9	3829352	c.2614G>C, p.D872H	2	0.0000071	1	0.22	LOF
9	3829343	c.2623C>T, p.H875Y	0	0	1	0.36	LOF
9	3829330	c.2636C>T, p.S879L	38	0.00013	2	0.27	LOF
9	3829330	c.2636C>G, p.S879W	0	0	1	0.37	LOF
9	3829318	c.2648G>C, p.G883A	0	0	1	0.28	LOF

9	3828360	c.2705G>A, p.R902H	9	0.000036	1	0.032	Neutral
9	3828355	c.2710G>A, p.G904R	58	0.00021	18	0.17	LOF
9	3828324	c.2741G>A, p.S914N	0	0	1	0.074	Neutral
9	3828319	c.2746G>A, p.V916M	38	0.00013	3	0.026	Neutral
9	3828316	c.2747_2749del, p.V916del	0	0	1	NA	LOF
9	3828316	c.2749G>A, p.D917N	0	0	1	0.21	LOF
9	3828295	c.2770T>G, p.S924A	0	0	1	0.23	LOF
9	3828280	c.2785G>A, p.E929K	3	0.000011	1	0.24	LOF

[a]According to REVEL prediction tool (pathogenicity threshold of 50%) [7].

[b]According to our *in vitro* luciferase assays.

Chr, chromosome; ***GnomAD***, genome aggregation database browser (v2.1.1); ***LOF***, loss-of-function; ***MAC***, minor allele count; ***MAF***, minor allele frequency; ***Pos***, position (according to the human alignment hg19/GRCh37).

ESM Table 2. ACMG criteria for the 105 rare coding variants of *GLIS3* (NM_001042413.2).

Mutation	PVS1	PS3	PM2	PM4	PP3	P/LP
c.34C>T, p.R12W	0	0	0	0	0	**0**
c.64A>G, p.M22V	0	0	0	0	0	**0**
c.82A>G, p.I28V	0	0	0	0	0	**0**
c.86C>T, p.P29L	0	0	1	0	0	**0**
c.106G>A, p.G36R	0	1	0	0	0	**0**
c.113C>G, p.P38R	0	0	0	0	0	**0**
c.202A>G, p.M68V	0	0	0	0	0	**0**
c.232C>G, p.R78G	0	0	0	0	0	**0**
c.233G>A, p.R78H	0	0	0	0	0	**0**
c.252A>T, p.L84F	0	0	0	0	0	**0**
c.256C>T, p.P86S	0	0	1	0	0	**0**
c.275C>T, p.T92I	0	0	0	0	0	**0**
c.287C>T, p.P96L	0	0	0	0	0	**0**
c.358G>C, p.G120R	0	1	0	0	0	**0**
c.370C>T, p.P124S	0	0	0	0	0	**0**
c.413G>A, p.C138Y	0	1	1	0	0	**1**
c.422T>C, p.I141T	0	1	0	0	0	**0**
c.425G>A, p.G142E	0	0	1	0	0	**0**
c.467T>G, p.M156R	0	1	1	0	1	**1**
c.472G>T, p.V158F	0	1	0	0	0	**0**
c.496C>G, p.P166A	0	1	0	0	0	**0**
c.503C>G, p.A168G	0	1	1	0	0	**1**
c.515C>T, p.S172F	0	1	1	0	0	**1**
c.713C>G, p.S238C	0	1	0	0	0	**0**
c.758G>C, p.S253T	0	0	1	0	0	**0**
c.793G>T, p.V265F	0	0	0	0	0	**0**
c.814T>A, p.Y272N	0	0	0	0	0	**0**
c.827C>T, p.T276M	0	1	0	0	0	**0**
c.833G>A, p.S278N	0	0	1	0	0	**0**
c.842C>T, p.S281F	0	1	1	0	0	**1**
c.844C>G, p.P282A	0	0	0	0	0	**0**
c.890G>A, p.R297H	0	0	0	0	0	**0**

c.893C>A, p.S298Y	0	1	0	0	0	**0**
c.937G>A, p.G313R	0	1	0	0	0	**0**
c.968C>T, p.S323L	0	1	0	0	1	**0**
c.974C>T, p.T325M	0	1	0	0	0	**0**
c.1012C>T, p.P338S	0	0	1	0	0	**0**
c.1063C>T, p.R355C	0	1	0	0	0	**0**
c.1073G>A, p.C358Y	0	1	1	0	0	**1**
c.1085C>A, p.P362Q	0	1	0	0	0	**0**
c.1090C>T, p.P364S	0	0	0	0	0	**0**
c.1111G>T, p.G371C	0	1	1	0	0	**1**
c.1117C>G, p.L373V	0	1	0	0	0	**0**
c.1126C>T, p.P376S	0	0	0	0	0	**0**
c.1145C>A, p.P382Q	0	0	0	0	0	**0**
c.1154G>T, p.G385V	0	0	0	0	0	**0**
c.1185G>A, p.M395I	0	0	1	0	0	**0**
c.1191G>C, p.Q397H	0	0	0	0	0	**0**
c.1199A>G, p.H400R	0	0	0	0	0	**0**
c.1200C>G, p.H400Q	0	0	0	0	0	**0**
c.1210C>A, p.Q404K	0	1	0	0	0	**0**
c.1214C>T, p.P405L	0	1	0	0	0	**0**
c.1216G>T, p.G406C	0	1	0	0	0	**0**
c.1227C>A, p.N409K	0	1	0	0	0	**0**
c.1232T>A, p.M411K	0	0	1	0	0	**0**
c.1271C>G, p.S424W	0	1	1	0	0	**1**
c.1295G>A, p.R432H	0	1	1	0	0	**1**
c.1318A>G, p.T440A	0	0	0	0	0	**0**
c.1325A>C, p.D442A	0	0	1	0	0	**0**
c.1336G>A, p.A446T	0	0	1	0	0	**0**
c.1388A>C, p.H463P	0	0	0	0	0	**0**
c.1417C>T, p.L473F	0	1	0	0	0	**0**
c.1430C>T, p.A477V	0	0	0	0	0	**0**
c.1480G>A, p.D494N	0	0	0	0	0	**0**
c.1483G>A, p.G495S	0	0	0	0	0	**0**
c.1518C>G, p.D506E	0	1	0	0	0	**0**
c.1545G>C, p.E515D	0	0	0	0	0	**0**
c.1709C>T, p.T570M	0	0	0	0	0	**0**

c.1715A>T, p.E572V	0	1	0	0	1	**0**
c.1775C>G, p.T592R	0	1	0	0	1	**0**
c.1843G>A, p.A615T	0	0	0	0	0	**0**
c.1881T>A, p.Y627*	1	1	1	0	0	**1**
c.1994G>A, p.S665N	0	0	0	0	0	**0**
c.2051C>T, p.P684L	0	0	0	0	0	**0**
c.2060C>T, p.S687F	0	1	0	0	0	**0**
c.2067A>T, p.R689S	0	0	1	0	0	**0**
c.2089G>A, p.V697M	0	1	0	0	0	**0**
c.2096G>A, p.R699H	0	0	0	0	0	**0**
c.2107C>T, p.P703S	0	0	0	0	0	**0**
c.2141C>G, p.S714C	0	0	0	0	0	**0**
c.2281G>A, p.D761N	0	1	0	0	0	**0**
c.2305C>A, p.P769T	0	0	0	0	0	**0**
c.2312C>A, p.A771D	0	0	1	0	0	**0**
c.2404G>A, p.G802S	0	0	0	0	0	**0**
c.2431G>C, p.E811Q	0	1	0	0	0	**0**
c.2439C>G, p.N813K	0	1	1	0	0	**1**
c.2461A>G, p.I821V	0	0	1	0	0	**0**
c.2494A>G, p.K832E	0	0	0	0	0	**0**
c.2518G>A, p.D840N	0	1	0	0	0	**0**
c.2531T>C, p.I844T	0	0	0	0	0	**0**
c.2549C>G, p.S850C	0	1	1	0	0	**1**
c.2609G>A, p.S870N	0	0	0	0	0	**0**
c.2614G>C, p.D872H	0	1	0	0	0	**0**
c.2623C>T, p.H875Y	0	1	1	0	0	**1**
c.2636C>T, p.S879L	0	1	0	0	0	**0**
c.2636C>G, p.S879W	0	1	1	0	0	**1**
c.2648G>C, p.G883A	0	1	1	0	0	**1**
c.2705G>A, p.R902H	0	0	0	0	0	**0**
c.2710G>A, p.G904R	0	1	0	0	0	**0**
c.2741G>A, p.S914N	0	0	1	0	0	**0**
c.2746G>A, p.V916M	0	0	0	0	0	**0**
c.2747_2749del, p.V916del	0	1	1	1	0	**1**
c.2749G>A, p.D917N	0	1	1	0	0	**1**
c.2770T>G, p.S924A	0	1	1	0	0	**1**

c.2785G>A, p.E929K	0	1	0	0	0	**0**

"1" means that the variant meets the ACMG criterion mentioned above the column; "0" means that the variant does not meet the ACMG criterion. ***PVS1,*** very strong pathogenic criterion #1; ***PS3,*** strong pathogenic criterion #3 (according to our *in vitro* assays); ***PM2,*** moderate pathogenic criterion #2 (according to GnomAD [v2.1.1]); ***PM4,*** moderate pathogenic criterion #4; ***PP3,*** supporting pathogenic criterion #3 (according to REVEL prediction tool); ***P/LP***, pathogenic or likely pathogenic.

ESM Table 3. Assessment of rare pathogenic/likely pathogenic variant enrichment in *GLIS3* coding exons.

Exons pair	Number of amino acids	Number of P/LP variants	P/LP variants: amino acids ratio (‰)	P/LP variants: amino acids ratio Exons 9-10 pair *vs* other exon pair	
				OR [95% CI]	*p*-value
**Exons 1-2 pair**	199	4	20.1	3.5 [3.0-4.0]	1.2×10^{-6}
**Exons 3-4 pair**	425	5	11.8	5.9 [5.3-6.5]	2.0×10^{-8}
**Exons 5-6 pair**	86	1	11.6	5.9 [5.3-6.5]	2.0×10^{-8}
**Exons 7-8 pair**	115	1	8.7	7.9 [7.2-8.6]	7.6×10^{-9}
**Exons 9-10 pair**	105	7	66.7	-	-

Pathogenic and likely pathogenic variant enrichment in *GLIS3* coding exons was assessed using a non-adjusted logistic regression model (P/LP variant ~ exon pair after accounting for exon length). ***CI***, confidence interval; ***OR***, odds ratio; ***P/LP***, pathogenic/likely pathogenic; ***vs***, versus.

ESM Table 4. Association analyses between pathogenic or likely pathogenic *GLIS3* variants and metabolic traits among patients with type 2 diabetes.

Trait	*N*	Effect size (SE)	$P_{overall}$
**BMI**	2,172	0.38 (1.9)	0.43
**Age of T2D diagnosis**	2,164	1.1 (2.2)	0.47
**HDL**	2,104	0.013 (0.11)	0.29
**Total cholesterol**	2,141	0.18 (0.35)	0.90
**LOG_TG**	2,129	-0.031 (0.15)	0.70

The association studies between the burdens of variants (considered as a single cluster) and clinical traits were performed using the mixed-effects score test (MiST), and were adjusted for age, sex, BMI and ancestry (for assessing type 2 diabetes risk, age at type 2 diabetes diagnosis, cholesterol levels), or for age, sex and ancestry (for assessing BMI). ***HDL***, high density lipoprotein; ***TG***, triglycerides; ***T2D***: type 2 diabetes.

ESM Table 5. Pathogenic or likely pathogenic mutations of *GLIS3* (NM_001042413.2) detected in 52K and TOPMed studies.

Chr	Pos (Hg38)	Mutation	TopMED	52K
9	4286332	c.94C>T, p.Arg32Ter	x	x
9	4286286	c.140C>A, p.Ser47Ter		x
9	4286230	c.196G>T, p.Gly66Ter		x
9	4286143	c.282del, p.Lys95SerfsTer18		x
9	4286137	c.289C>T, p.Arg97Ter	x	
9	4286097	c.328dup, p.His110ArgfsTer72	x	
9	4286037	c.388+1G>A	x	
9	4125852	c.478C>T, p.Arg160Ter	x	x
9	4125801	c.529C>T, p.Gln177Ter		x
9	4118240	c.1237del, p.Val413CysfsTer29	x	
9	4118198	c.1276_1279dup, p.Leu427ArgfsTer128	x	
9	4117945	c.1533C>G, p.Tyr511Ter		x
9	3879566	c.2158C>T, p.Arg720Ter		x
9	3828409	c.2657-1G>C	x	

Among these variants, 7 pathogenic or likely pathogenic variants per study were kept for further association analysis with type 2 diabetes risk.

ESM Figure 1. Functional activity of rare *GLIS3* variants, according to *in vitro* luciferase assays.

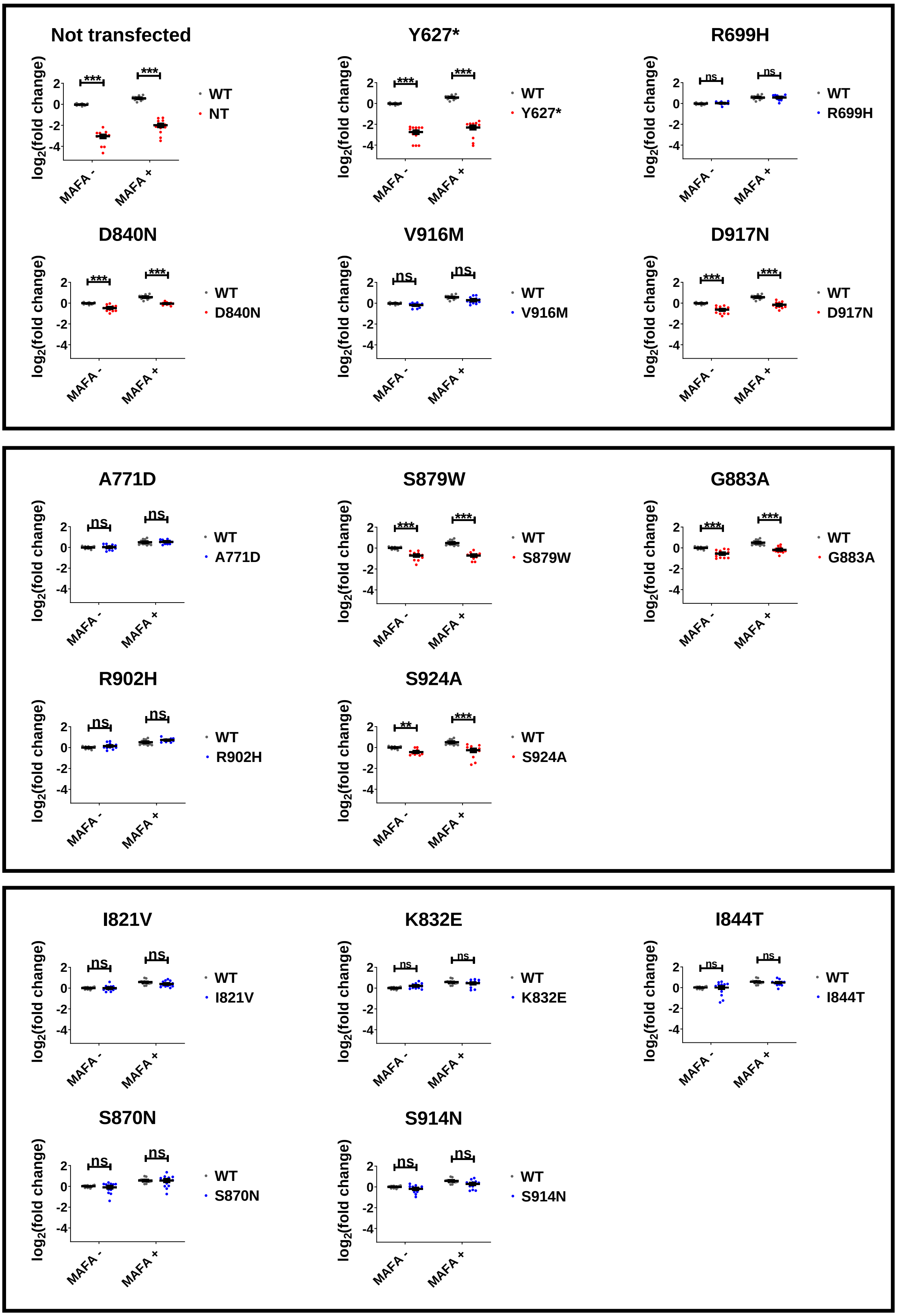

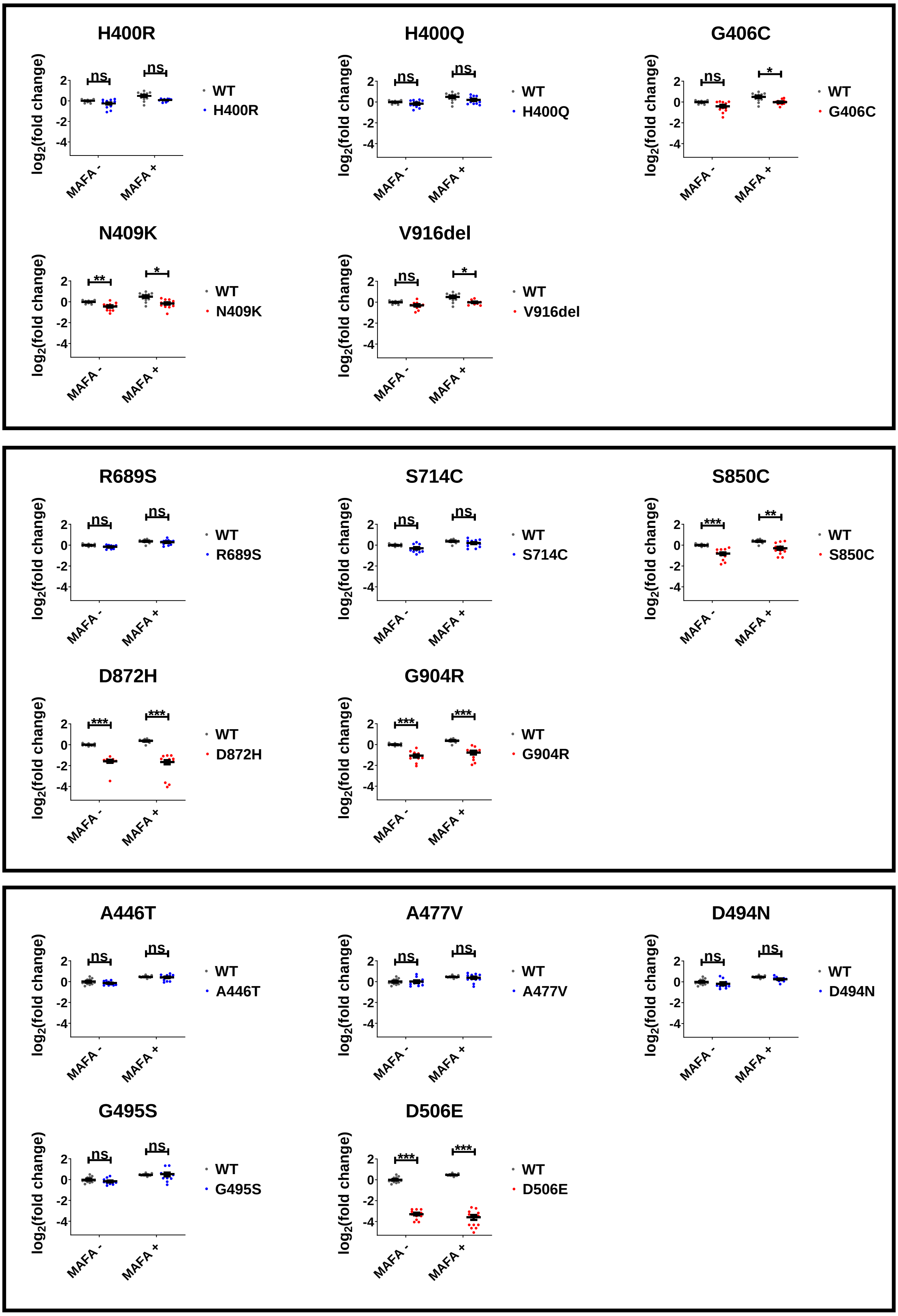
H400R
ns
ns
WT
H400R
H400Q
ns
ns
WT
H400Q
G406C
ns
*
WT
G406C
N409K
**
*
WT
N409K
V916del
ns
*
WT
V916del
R689S
ns
ns
WT
R689S
S714C
ns
ns
WT
S714C
S850C
***
**
WT
S850C
D872H
***
***
WT
D872H
G904R
***
***
WT
G904R
A446T
ns
ns
WT
A446T
A477V
ns
ns
WT
A477V
D494N
ns
ns
WT
D494N
G495S
ns
ns
WT
G495S
D506E
***
***
WT
D506E
log_2(fold change)
2
0
-2
-4
MAFA -
MAFA +

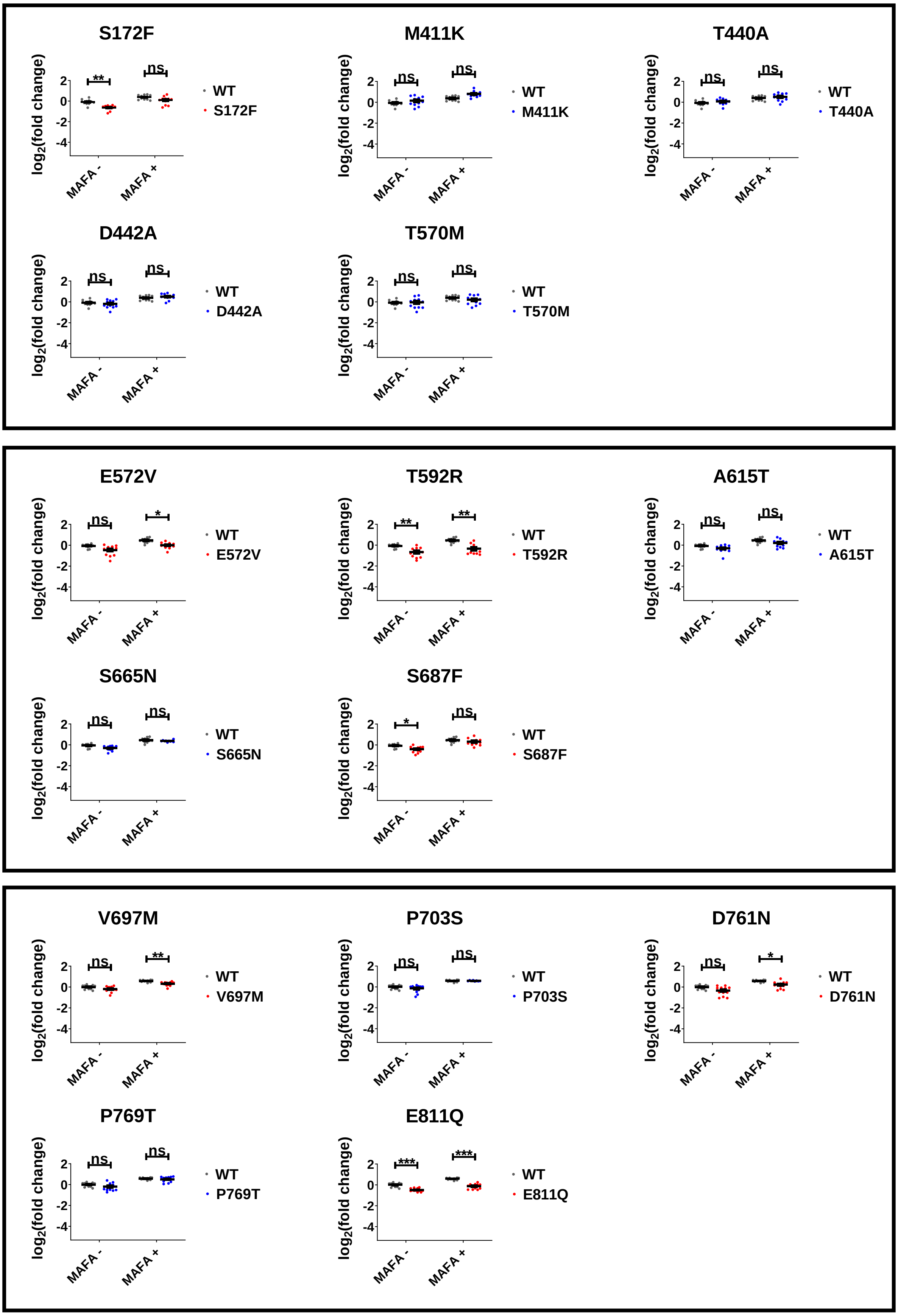

S172F
**
ns
WT
S172F
M411K
ns
ns
WT
M411K
T440A
ns
ns
WT
T440A
D442A
ns
ns
WT
D442A
T570M
ns
ns
WT
T570M
E572V
ns
*
WT
E572V
T592R
**
**
WT
T592R
A615T
ns
ns
WT
A615T
S665N
ns
ns
WT
S665N
S687F
*
ns
WT
S687F
V697M
ns
**
WT
V697M
P703S
ns
ns
WT
P703S
D761N
ns
*
WT
D761N
P769T
ns
ns
WT
P769T
E811Q
***
***
WT
E811Q
log2(fold change)
2
0
-2
-4
MAFA -
MAFA +

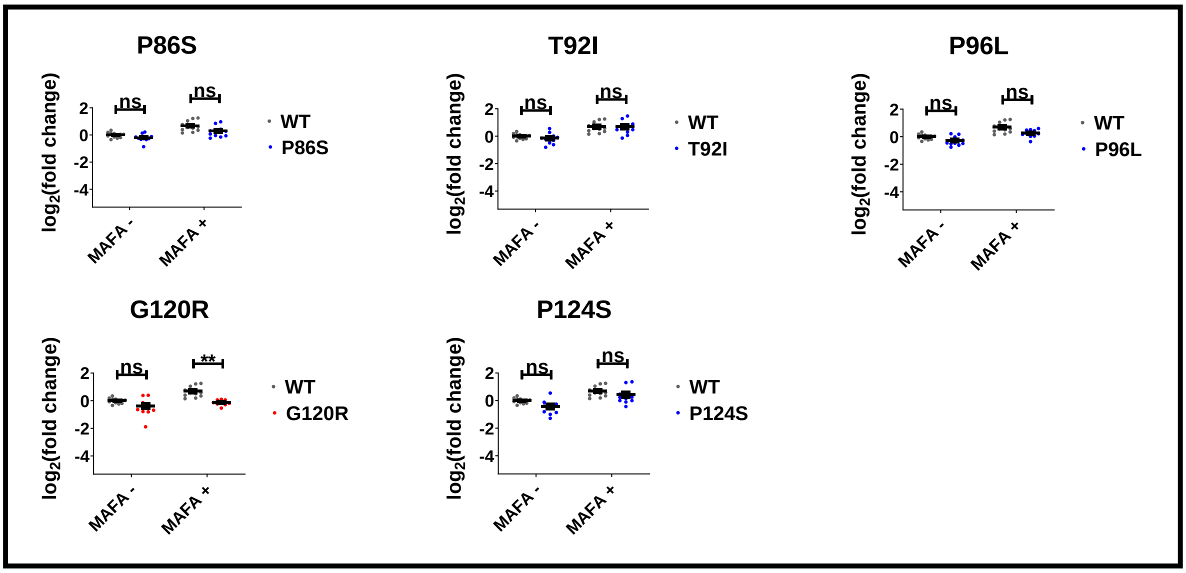
P86S
T92I
P96L
G120R
P124S
log2(fold change)
MAFA -
MAFA +
ns
**
WT
P86S
T92I
P96L
G120R
P124S

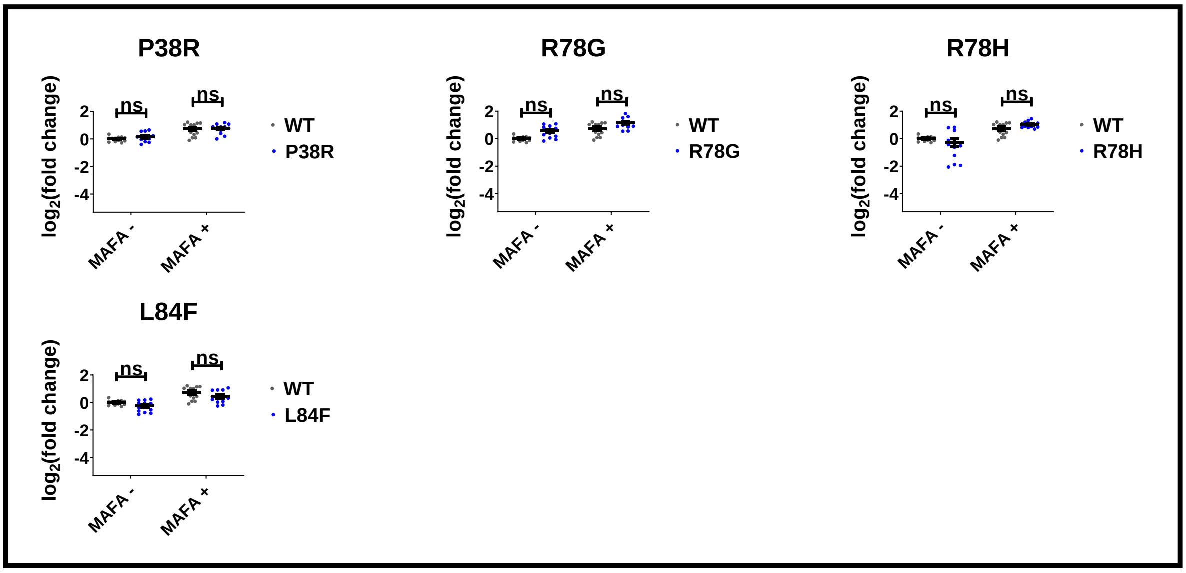
P38R
R78G
R78H
L84F
log2(fold change)
MAFA -
MAFA +
ns
WT
P38R
R78G
R78H
L84F

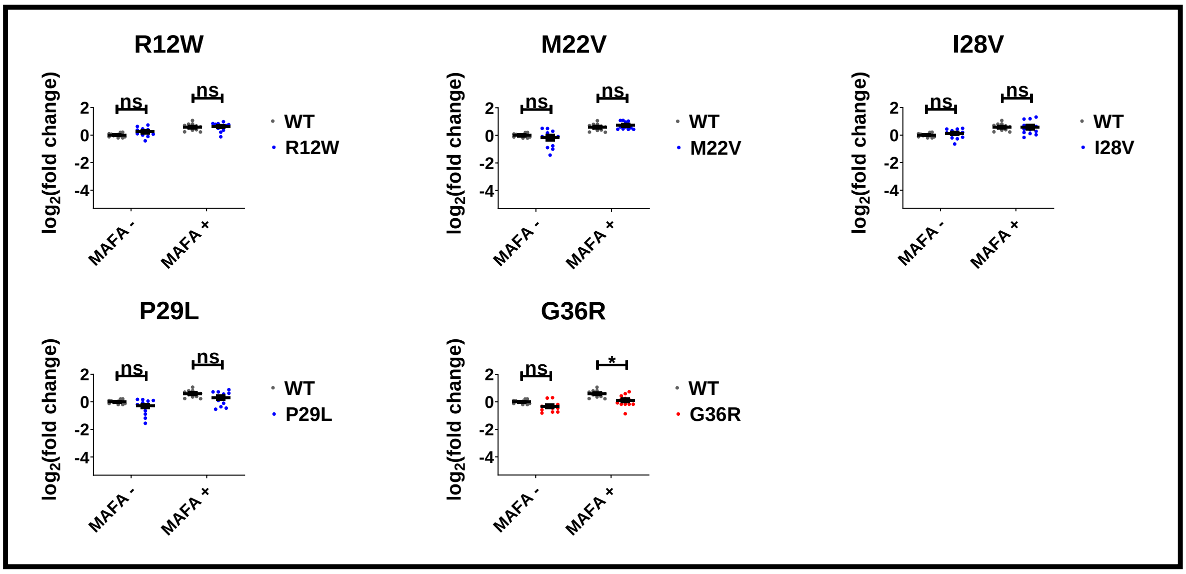
R12W
M22V
I28V
P29L
G36R
log2(fold change)
MAFA -
MAFA +
ns
*
WT
R12W
M22V
I28V
P29L
G36R

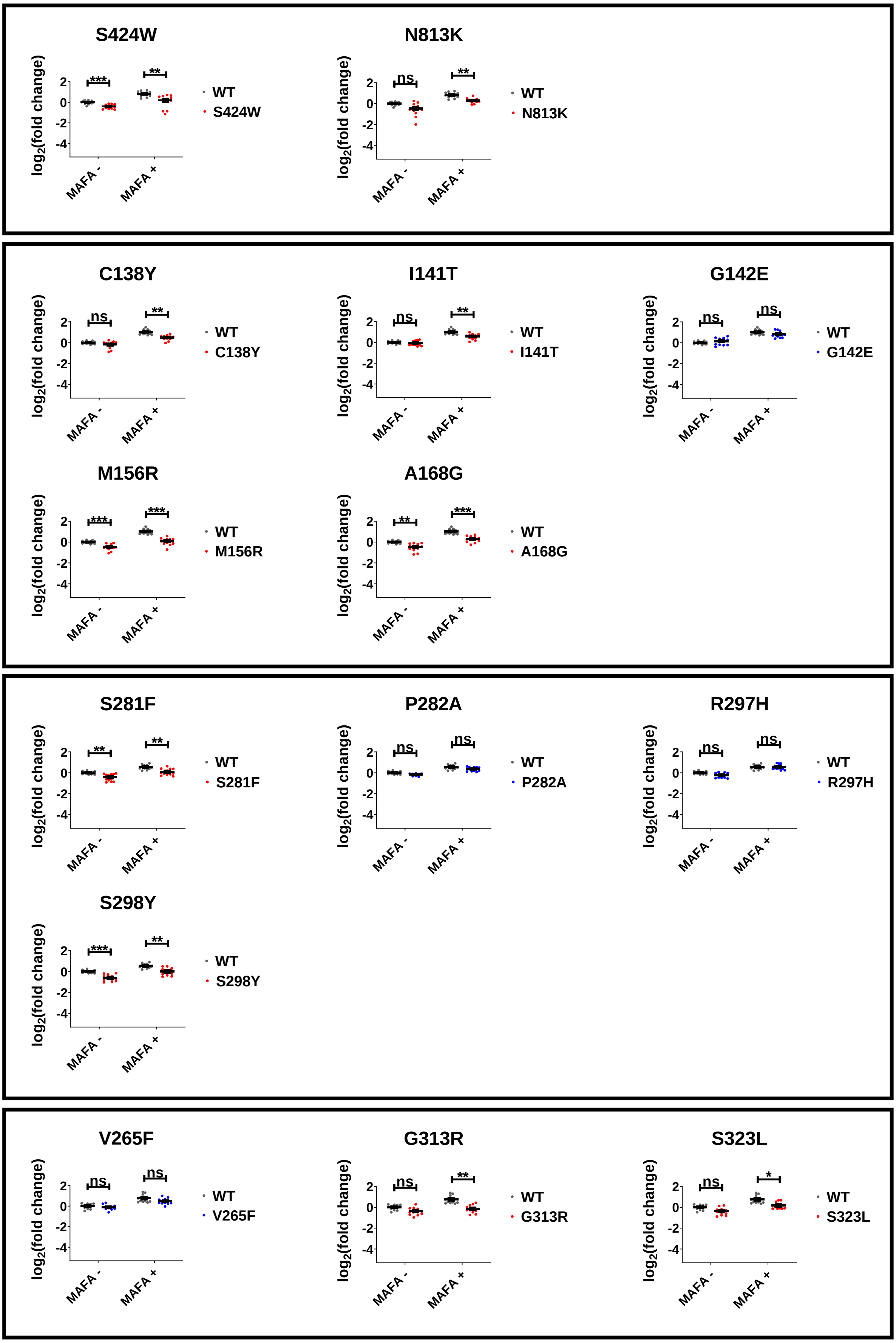
S424W
N813K
C138Y
I141T
G142E
M156R
A168G
S281F
P282A
R297H
S298Y
V265F
G313R
S323L
log2(fold change)
MAFA -
MAFA +
ns
*
**
***
WT
S424W
N813K
C138Y
I141T
G142E
M156R
A168G
S281F
P282A
R297H
S298Y
V265F
G313R
S323L

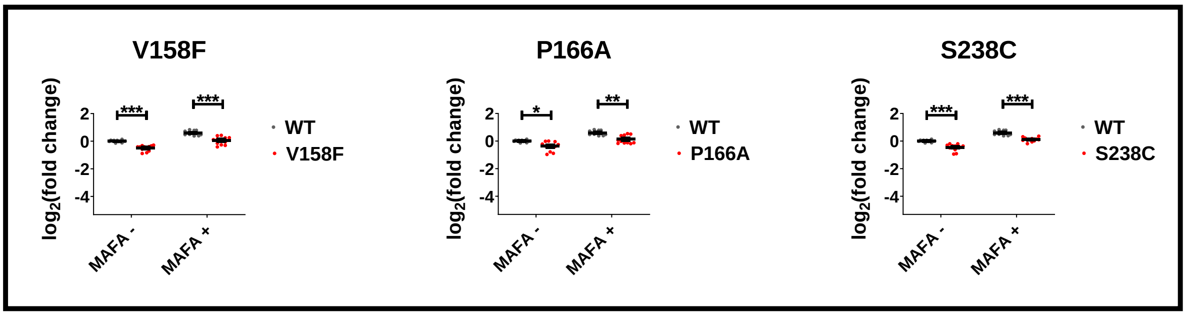
V158F
log2(fold change)
***
***
WT
V158F
P166A
*
**
WT
P166A
S238C
***
***
WT
S238C
MAFA -
MAFA +

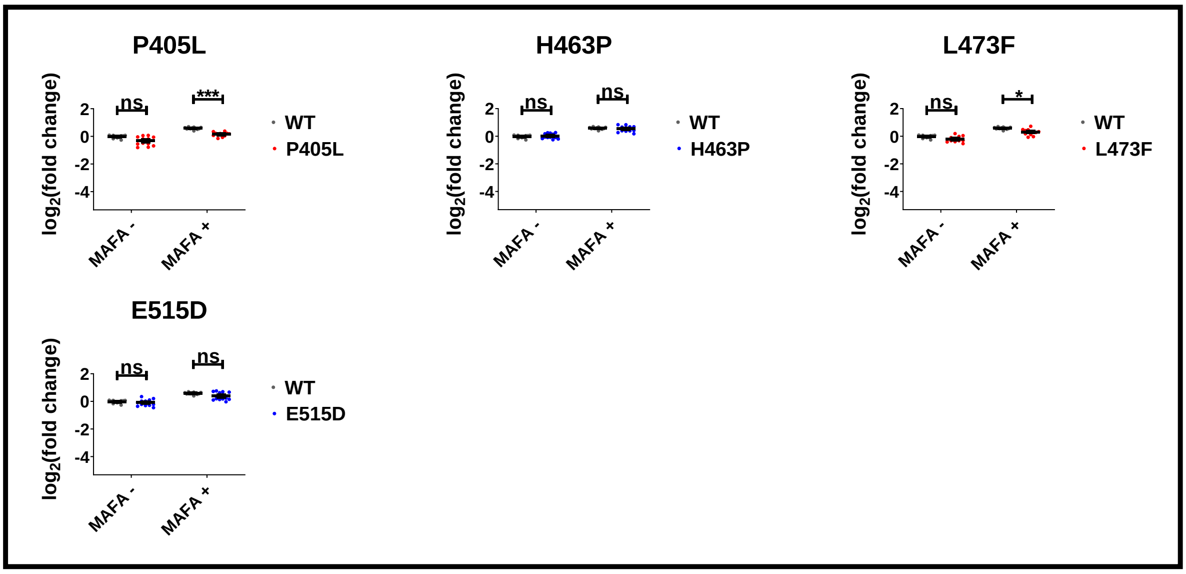
P405L
ns
***
WT
P405L
H463P
ns
ns
WT
H463P
L473F
ns
*
WT
L473F
E515D
ns
ns
WT
E515D
log2(fold change)
MAFA -
MAFA +

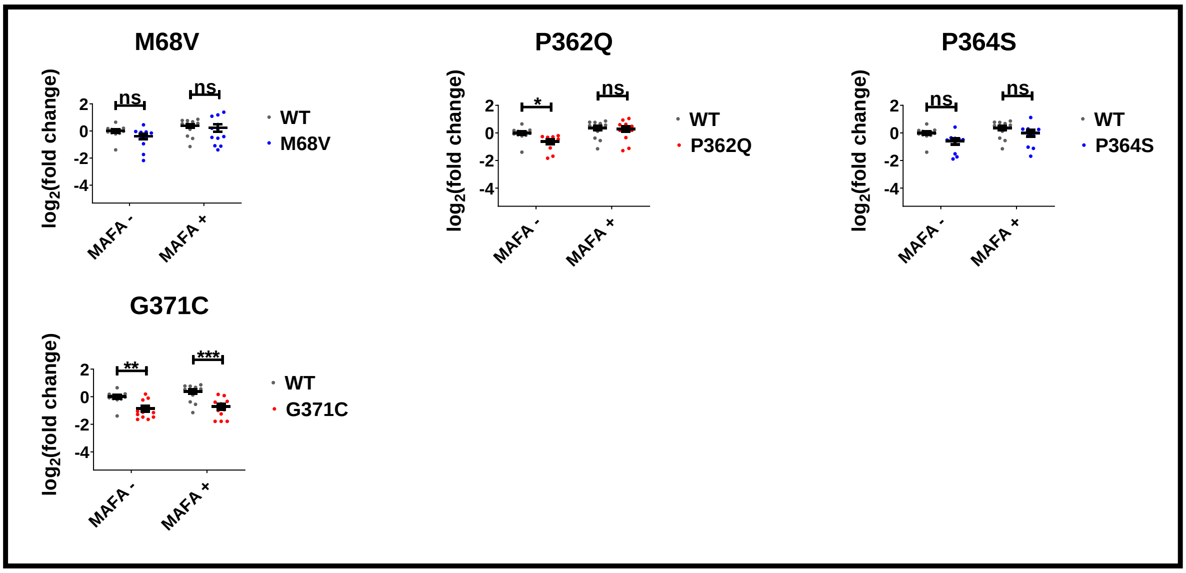
M68V
ns
ns
WT
M68V
P362Q
*
ns
WT
P362Q
P364S
ns
ns
WT
P364S
G371C
**
***
WT
G371C
log2(fold change)
MAFA -
MAFA +

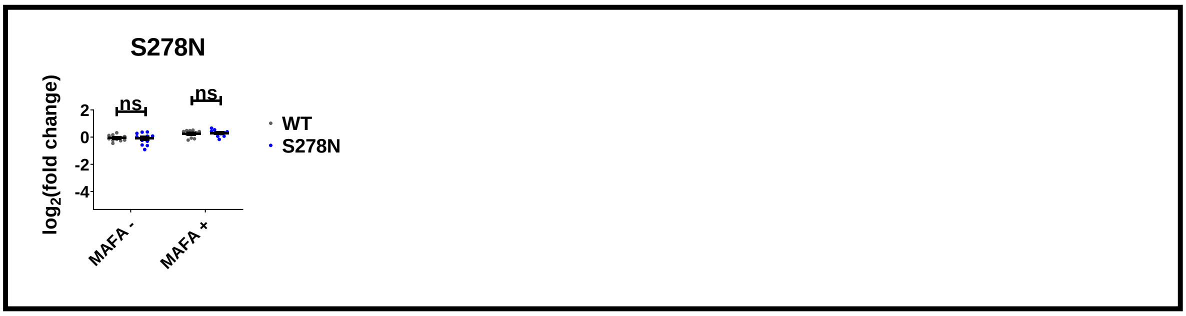
S278N
ns
ns
WT
S278N
log2(fold change)
MAFA -
MAFA +

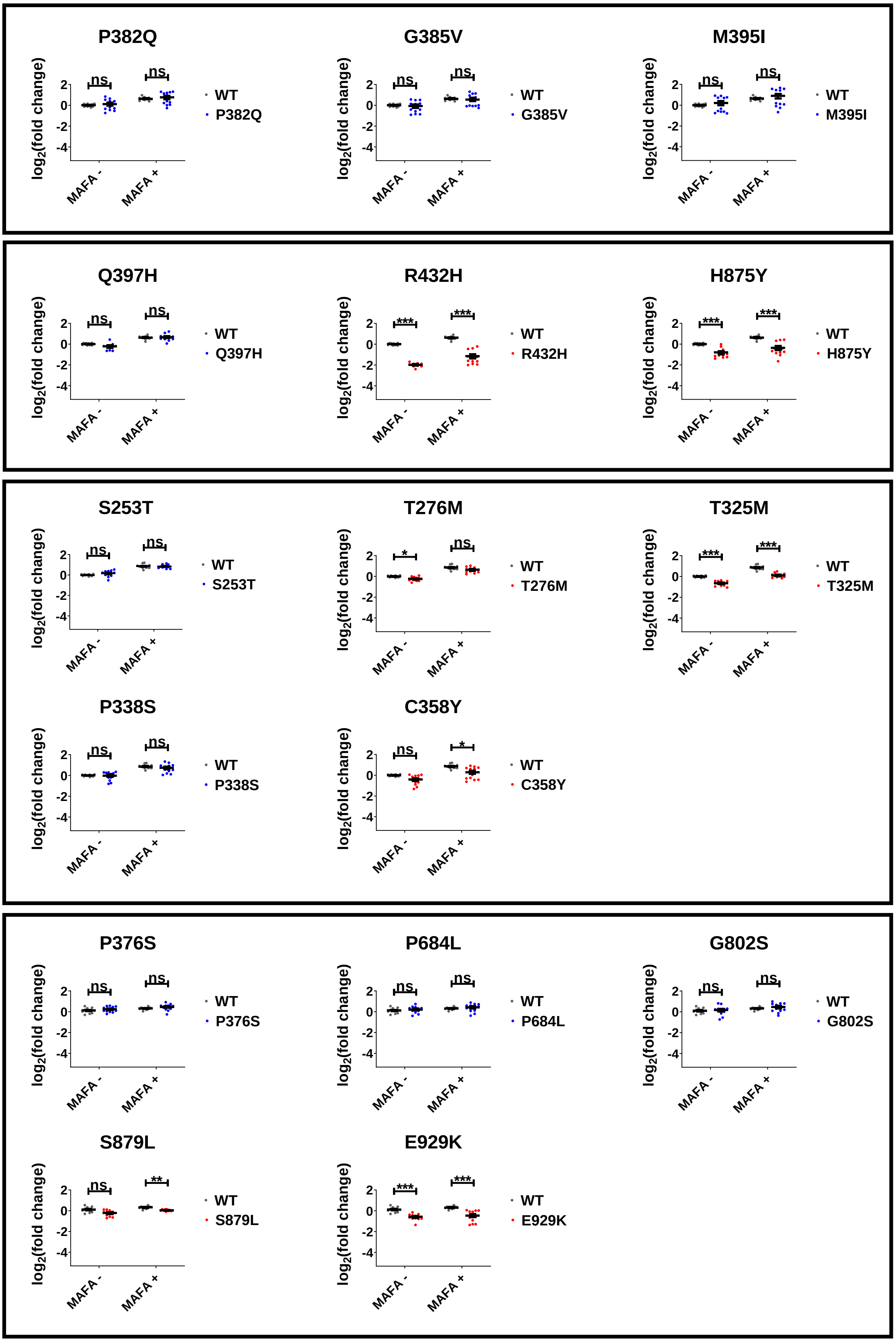

P382Q
log2(fold change)
2
0
-2
-4
MAFA -
MAFA +
ns
ns
WT
P382Q
G385V
ns
ns
WT
G385V
M395I
ns
ns
WT
M395I
Q397H
ns
ns
WT
Q397H
R432H
***
***
WT
R432H
H875Y
***
***
WT
H875Y
S253T
ns
ns
WT
S253T
T276M
*
ns
WT
T276M
T325M
***
***
WT
T325M
P338S
ns
ns
WT
P338S
C358Y
ns
*
WT
C358Y
P376S
ns
ns
WT
P376S
P684L
ns
ns
WT
P684L
G802S
ns
ns
WT
G802S
S879L
ns
**
WT
S879L
E929K
***
***
WT
E929K

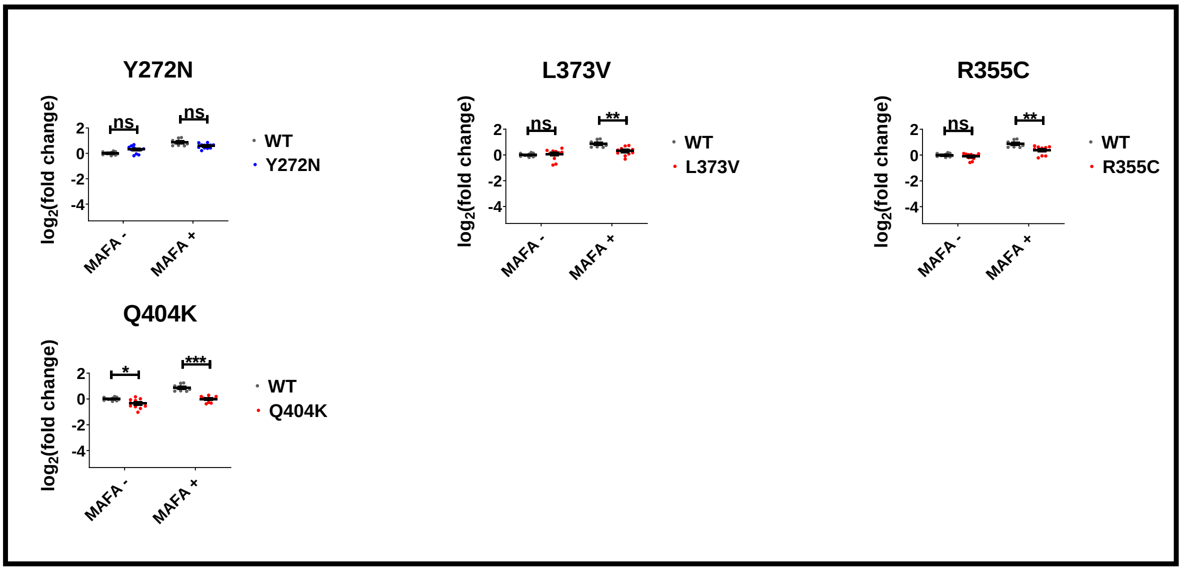

The figure shows the log2(fold change) (relative to the wild-type *GLIS3*) of luciferase activity in HEK293 cells transfected (or not) with wild type or mutant *GLIS3*. Loss-of-function variants are represented in red, neutral variants are represented in blue. Variants tested simultaneously on the same plate and using the same control condition were clustered together. Data represent the mean ± SEM of the fold change from at least four independent experiments performed in technical triplicate. *P < 0.05; **P < 0.01; ***P < 0.001 (*versus* wild-type *GLIS3*). ***WT***, wild-type; ***NT***, not transfected; ***ns***, not significant.
